# Supplementary material for: Content-rich biological network constructed by mining PubMed abstracts
Source: BMC Bioinformatics. 2004 Oct 8;5:147. doi: 10.1186/1471-2105-5-147 (PMC528731; doi:10.1186/1471-2105-5-147)
Supplement: Additional File 5 — The original Chilibot query results of the term "long-term potentiation (LTP)" and 22 other terms, limiting the latest references analyzed to the years 1990, 1995, 2000, and 2004. [file 1471-2105-5-147-S5.bz2 › chilibotAdditionalFile5/ltp2004/html/PKC_SYNAPTOTAGMIN.html]

 


 **PKC** and **SYNAPTOTAGMIN** 
  
Found 14 abstracts in PubMed,  **14 abstracts were retrieved and analyzed**.  


---

 Search Google  |
 PDF files only 
|  EDU domain only 

---

**Interactive relationship** (e.g. stimulation, inhibition, etc)

- SyntagI, the purified, recombinant, cytoplasmic domain of rat  **synaptotagmin**  I, was an effective substrate in vitro for calcium calmodulin dependent protein kinase II CaMKII, protein kinase C  **PKC** , and casein kinase II caskII.  Ref: 10461881 J Neurochem, 1999

**Parallel relationship** (e.g. studied together, co-existance, homology, etc.)

- The results suggest the possibility that the phosphorylation of  **synaptotagmin**  I by CaMKII and  **PKC**  contributes to the mechanism s by which these two kinases regulate neurotransmitter release.  Ref: 10461881 J Neurochem, 1999
- Models of the two zinc binding modules of  **PKC**  alpha are based on nuclear magnetic resonance and crystal structures of such modules in other  **PKC**  isoforms while the calcium phospholipid binding module C2 is based on the crystal structure of a repeating unit in  **synaptotagmin**  I.  Ref: 8916229 Proteins, 1996
- In this study, we quantitatively compared the equilibrium and kinetic parameters of C2 domains isolated from three functionally distinct signaling proteins cytosolic phospholipase A2 alpha cPLA2 alpha, protein kinase C beta  **PKC**  beta, and  **synaptotagmin**  IA Syt IA.  Ref: 11258923 Biochemistry, 2001
